# The Puzzle of Argument Structure Mismatch in Gapping

**DOI:** 10.3389/fpsyg.2022.907823

**Published:** 2022-05-20

**Authors:** Jiayi Lu, Nayoun Kim

**Affiliations:** ^1^Department of Linguistics, Stanford University, Stanford, CA, United States; ^2^Department of English Language and Literature, Sungkyunkwan University, Seoul, South Korea

**Keywords:** gapping, argument structure mismatch, parallelism, locative alternation, dative alternation

## Abstract

Voice mismatch between conjuncts is impossible in the gapping construction. Some recent studies explained this effect by analyzing gapping as involving the ellipsis of a category at least as large as VoiceP. One prediction this analysis makes is that mismatch of any head structurally lower than Voice (e.g., little v) should not be possible in gapping. In this study, through a series of acceptability judgment experiments examining argument structure mismatches in gapping, we provide empirical observations that challenge this prediction.

## Introduction

When the same verb is shared across conjuncts in a coordinate structure, the verbs in the non-initial conjuncts can sometimes be removed, yielding the “Gapping” construction (Jackendoff, 1971; Johnson, 1996, 2006; Chaves, 2005; Yoshida et al., 2012). An example is shown in (1).

(1) John finished the cake, and Mary the brownies.

Merchant (2008, 2013), among others, noted that gapping cannot be licensed when there is a voice mismatch between the conjuncts. As in the ungrammatical examples in (2), when one conjunct is in the active voice and the other conjunct is in the passive voice, gapping is not possible. In contrast, the non-elliptical counterparts to the sentences in (2), shown in (3), are totally fine.

(2) a. *Some bring roses and lilies by others.
     b. *Lilies are brought by some and others roses.

(Merchant, 2013, p. 83)

(3) a. Some bring roses and lilies are brought by others.
     b. Lilies are brought by some and others bring roses.

(Merchant, 2013, p. 84)

To account for the ungrammaticality of sentences in (2), Merchant (2013) proposed that gapping involves an ellipsis larger than vP. Merchant (2013) assumed an English clause structure as shown below in (4), following Collins (2005). The Voice head, which projects a VoiceP that is situated between TP and vP, is responsible for the active–passive voice distinction (for other instances of parallelism requirement in ellipsis, refer to Merchant, 2008).

(4) [_TP_…T [_VoiceP_…Voice [_vP_… v [_VP_… V …]]]]

Assuming that an identity requirement of ellipsis is calculated over syntactic structure and features (Sag, 1976; Fiengo and May, 1994; Chung et al., 1995; Baltin, 2012), the value of the elided Voice head needs to match the value of the antecedent Voice head. However, in sentences such as (2a) and (2b), the antecedent Voice head and the elided Voice head mismatch in value: one is passive, and the other is active. The identity requirement of ellipsis is not satisfied, and the resulting sentences are ungrammatical. Similar to gapping, other constructions that involve an ellipsis of categories larger than VoiceP also do not tolerate active–passive mismatch. Under Merchant’s (2008) analysis, these constructions include sluicing, stripping, pseudogapping, and fragment answers (Johnson, 1996, 2004; Merchant, 2001).

One natural prediction is that, when the height of the ellipsis is high enough to disallow Voice mismatch, mismatch in anything structurally lower than VoiceP should also be disallowed. Merchant (2013) discussed the ungrammaticality of argument structure alternations (e.g., dative alternation and locative alternation) in sluicing, which has been widely attested previously (Chung et al., 1995; Merchant, 2001; Chung, 2006), as the validation of this prediction. Consider (5).

(5) *They served someone something, but I don’t know what to whom. Adapted from Chung (2006)

Assuming the clause structure in (4) and that the two argument structures in dative alternation involve mismatch in little v, the ungrammaticality of (5) suggests that little v mismatch is impossible in sluicing, confirming the earlier prediction. If gapping, just like sluicing, also involves the ellipsis of a category at least as large as VoiceP [as shown by the contrast between (2) and (3)], little v mismatch in gapping should also be ungrammatical. However, it is unclear whether this is empirically accurate. Consider (6), which is the analog of (5) with gapping.

(6) ?Austin promised the team a banquet, and Sydney a bonus to the crew.

Sentence (6) involves a gapping construction. The two conjuncts in (6) mismatch in argument structure in the same way as in (5). Following all aforementioned assumptions, the two conjuncts in (6) involve little v mismatch. Although no judgment is given for examples like (6) in Merchant (2013), such sentences are expected to be ungrammatical as well because the identity requirement of ellipsis is violated. However, according to the informal judgments given by the native speakers we consulted, sentence (6) is only borderline degraded in acceptability, and it is unclear whether it should be marked as ungrammatical, such as (2) and (5), which share the same identity condition violation with (6) under Merchant’s (2013) analysis.

This study aims to experimentally verify the hypothesis that gapping does not allow argument structure mismatch between conjuncts. In a series of acceptability judgment experiments, we probe whether an acceptability penalty is induced by argument structure mismatch in gapping. Furthermore, if such a penalty does exist, we probe whether this effect is a specific property of gapping, or a property of coordinate structures in general. In Experiments 1a and 1b, we explored the aforementioned two questions using the locative alternation. In Experiments 2a and 2b, we explored the same set of questions using the dative alternation.

## Experiment 1a

This experiment aims to provide evidence for the argument structure mismatch penalty in gapping. Specifically, we examine gapping constructions with locative alternation verbs (e.g., *load* and *cram*) in the conjuncts. Since these verbs participate in the locative alternation and are compatible with two different argument structure frames, we can test whether argument structure mismatch is indeed disallowed in gapping.

### Method

#### Participant

In this experiment, 53 self-reported native speakers of English with no vision or hearing disorders were recruited through *Prolific.co*, a crowdsourcing platform. All participants provided informed consent and were compensated at a rate of approximately $10 per hour for their participation.

#### Materials

All critical items were of the gapping construction, with the main verb in both conjuncts being a locative alternation verb. Locative alternation verbs can take two internal arguments: a figure argument which is the moving object in the event, and a ground argument that indicates the location (Kim, 1999; Kim et al., 1999). These verbs display two different argument structures: *with*-frame, where the figure argument appears in a prepositional phrase (PP; usually headed by *with*, hence the name “with-frame”) and the ground arguments appear as a direct object; and *in*-frame, where the figure argument appears as the direct object and the ground argument appears in a PP (usually headed by *in*, hence the name “in-frame,” but other prepositions like *on* and *onto* may also appear). Examples (7a) and (7b) show the *with*-frame and *in*-frame argument structures of the verb *load*, respectively.

(7) a. John loaded the truck with the boxes.
     b. John loaded the boxes onto the truck.

In Experiment 1a, 16 critical items were each instantiated as four conditions in a 2 × 2 factorial design, with argument structure matching (match vs. mismatch between the two conjuncts) and *second conjunct argument structure* (*in*-frame vs. *with*-frame) as factors. A list of sample stimuli is shown in (8). The *argument structure matching* factor was included to test our hypothesis that the matching between the argument structure of the verbs is assumed in gapping processing. The *second conjunct argument structure* factor was included to control for any potential incompatibility between a particular argument structure and ellipsis, independent of whether the argument structures of the two conjuncts matched.

(8) a. William loaded the boat with the cargo, and Lauren the truck with the sack.
   [Argument structure match/second conjunct *with*-frame]
   b. William loaded the cargo onto the boat, and Lauren the sack onto the truck.
   [Argument structure match/second conjunct *in*-frame]
   c. William loaded the cargo onto the boat, and Lauren the truck with the sack.
   [Argument structure mismatch/second conjunct *with*-frame]
   d. William loaded the boat with the cargo, and Lauren the sack onto the truck.
   [Argument structure mismatch/second conjunct *in*-frame]

If there was an argument structure mismatch penalty in gapping, the main effect of *argument structure matching* was expected where the mismatch condition was rated lower than the match condition. If there was no idiosyncratic incompatibility between any argument structure and ellipsis, no significant main effect of *second conjunct argument structure* or any interaction between the two factors was expected.

In addition to the critical items, there were also 27 grammatical fillers (mean rating = 5.49, SE = 0.14) and 27 ungrammatical fillers (mean rating = 3.15, SE = 0.18).

#### Procedure

The experiment was implemented on Ibex Farm, a web-based presentation platform (Drummond, 2013). Participants took part in the experiment remotely on their laptops *via* a link distributed through *Prolific.co*. Stimuli were presented one at a time. Participants were asked to read the sentences and rate how acceptable they sounded on a 7-point Likert scale (1 = *totally unacceptable* to 7 = *totally acceptable*). A total of six practice questions were given prior to the actual experiment.

### Results

**FIGURE 1:**
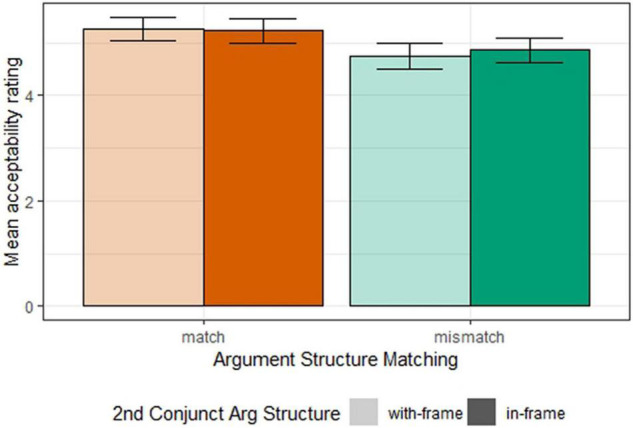
Mean acceptability ratings for all conditions (Experiment 1a).

Mean acceptability ratings for all conditions in Experiment 1a are shown in Figure 1. Data were analyzed with an ordinal regression model (Christensen and Christensen, 2015), predicting acceptability ratings with sum-coded fixed effects of *argument structure matching* (match vs. mismatch) and *second conjunct argument* (*in*-frame vs. *with*-frame) and their interactions, along with by-participant and by-item random intercepts and random slopes for the fixed effects and their interaction (Barr et al., 2013).

We found a significant effect of *argument structure matching* (β = 0.29, SE = 0.08, *z* = 3.67, *p* < 0.001) with the mismatching condition lower in acceptability than the matching condition. We found no significant effect of *second conjunct argument structure* (β = 0.02, SE = 0.07, *z* = −0.21, *p* = 0.83) or its interaction with *argument structure matching* (β = 0.05, SE = 0.07, *z* = 0.67, *p* = 0.50). This suggests that the two argument structures tested are equally compatible with the gapping construction, and the main effect of *argument structure matching* is not driven by any interaction between the factors.

The results suggest that, while the *with*-frame and *in*-frame are equally compatible with gapping, there is an argument structure mismatch penalty, i.e., when the two conjuncts display different argument structures, the resulting sentence is degraded in acceptability.

## Experiment 1b

This experiment tests whether the argument structure mismatch penalty observed in Experiment 1a is specific to gapping. To rule out the possibility that this phenomenon is not simply driven by the general preference for parallelism in coordination (Dubey et al., 2008; Sturt et al., 2010), we added the non-elliptical condition as a baseline.

### Method

#### Participants

In this experiment, participants were 65 self-reported native speakers of English with no vision or hearing disorders. They were recruited through *Prolific.co*, a crowdsourcing platform. Participants provided informed consent and were compensated at a rate of approximately $10 per hour.

#### Materials

A total of sixteen critical items were each instantiated as four conditions in a 2 × 2 factorial design, with *argument structure matching* (match vs. mismatch between the two conjuncts) and the *presence of gapping* (gapping vs. no gapping) manipulated as factors. A list of sample stimuli is shown in (9). The *argument structure matching factor* was included to test our hypothesis that the matching between the argument structure of the verbs is assumed in gapping processing. The *presence of gapping* factor was included to ensure that the penalty induced by the argument structure mismatch is confined to gapping construction.

(9) a. William loaded the cargo onto the boat, and Lauren the sack onto the truck.
   [Argument structure match/gapping]
   b. William loaded the cargo onto the boat, and Lauren loaded the sack onto the truck.
   [Argument structure match/no gapping]
   c. William loaded the cargo onto the boat, and Lauren the truck with the sack.
   [Argument structure mismatch/gapping]
   d. William loaded the cargo onto the boat, and Lauren loaded the truck with the sack.
   [Argument structure mismatch/no gapping]

If the argument structure mismatch penalty was specific to gapping, rather than a property of coordination in general, a significant interaction between *argument structure matching* and the *presence of gapping* was expected where the contrast between argument structure match and mismatch sentences was larger in the gapping condition than in the no gapping condition.

In addition to the critical items, there were also 16 grammatical fillers (mean rating = 4.94, SE = 0.10) included.

#### Procedure

The same experimental procedure as that of Experiment 1a was carried out.

### Results

**FIGURE 2:**
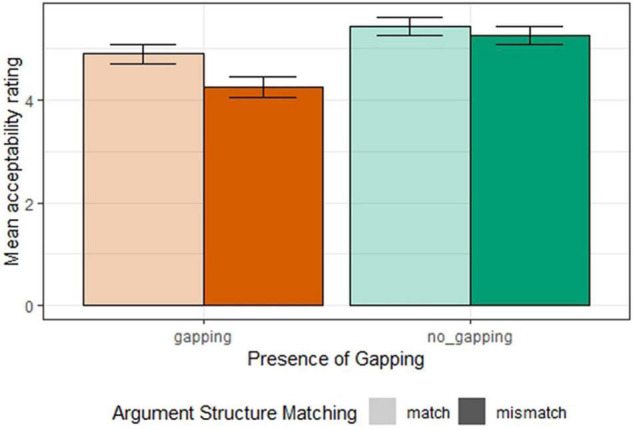
Mean acceptability ratings for all conditions (Experiment 1b).

Figure 2 shows the mean acceptability ratings for all conditions in Experiment 1b. The results were analyzed using an ordinal regression model predicting acceptability ratings with sum-coded fixed effects of *argument structure matching*, *second conjunct argument structure*, and their interaction, and random by-participant and by-item intercepts and slopes for the fixed effects and their interaction. We found a significant effect of *argument structure matching* (β = 0.29, SE = 0.07, *z* = 3.97, *p* < 0.001) with the mismatching condition lower in acceptability than the matching condition. We also found the main effect of the *presence of gapping* (β = −0.65, SE = 0.14, *z* = −4.60, *p* < 0.001), as well as an interaction between *argument structure matching* and the *presence of gapping* (β = 0.14, SE = 0.07, *z* = 1.97, *p* < 0.05). These results suggest that the argument structure matching penalty has a gapping-specific component and is not just a general property of coordination.

## Experiment 2a

This experiment aims to replicate the results from Experiment 1a using the dative alternation, instead of the locative alternation (Larson, 1988, 2017). The dative alternation verbs allow both the double object (DO) argument structure in (10a), and the PP argument structure in (10b). The two structures differ in how each thematic role is mapped onto the word order; in (10a), the NP denoting the recipient (*a team*) precedes the NP specifying the theme (*a banquet*), but the NP specifying the theme (*a banquet*) precedes the NP specifying the recipient (*a team*) in (10b).

(10) a. Austin promised [NP a team] [NP a banquet].
   b. Austin promised [NP a banquet] [PP to a team].

In this experiment, we used the same design as that of Experiment 1a to test, if the dative alternation argument structure mismatch is also disallowed in gapping.

### Method

#### Participants

In this experiment, 52 self-reported native speakers of English with no vision or hearing disorders participated on *Prolific.co*, a crowdsourcing platform. Participants provided informed consent and were compensated at a rate of approximately $10 per hour.

#### Materials

Same as in Experiment 1a, 16 critical items were each instantiated as four conditions in a 2 × 2 factorial design, with *argument structure matching* (match vs. mismatch) and *second conjunct argument structure* (DO-frame vs. *PP-frame*) manipulated as factors. A list of sample stimuli is shown in (11).

(11) a. Austin promised the team a banquet, and Sydney the crew a bonus.
  [Argument structure match/second conjunct DO-frame]
  b. Austin promised a banquet to the team, and Sydney a bonus to the crew.
  [Argument structure match/second conjunct PP-frame]
  c. Austin promised a banquet to the team, and Sydney the crew a bonus.
  [Argument structure mismatch/second conjunct DO-frame]
  d. Austin promised the team a banquet, and Sydney a bonus to the crew.
  [Argument structure mismatch/second conjunct PP-frame]

In addition to the critical items, there were 32 grammatical (mean rating = 4.75, SE = 0.10) and 32 ungrammatical fillers (mean rating = 2.63, SE = 0.08) included.

#### Procedure

The same experimental procedure as that of Experiment 1a was carried out.

### Results

**FIGURE 3:**
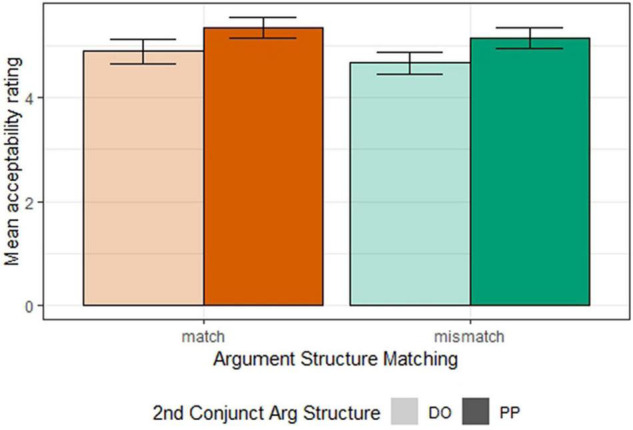
Mean acceptability ratings for all conditions (Experiment 2a).

Figure 3 shows the mean acceptability ratings for all conditions in Experiment 2a. The same ordinal regression model as that of Experiment 1a was used to analyze the results. We found a marginal effect of *argument structure matching* (β = 0.18, SE = 0.10, *z* = 1.92, *p* = 0.055) and a significant effect of *second conjunct argument structure* (β = −0.363, SE = 0.10, *z* = −3.47, *p* < 0.001), such that *DO*-frame was less compatible with gapping than the *PP*-frame. Furthermore, no interaction between the *argument structure matching* and *second conjunct argument structure* (β = 0.032, SE = 0.10, *z* = 0.33, *p* = 0.74) was observed. If the marginal *argument structure matching* effect is taken to be reliable, this suggests an argument structure mismatch penalty as that of Experiment 1a. However, when the second conjunct displays the *DO*-frame, gapping is degraded regardless of the argument structure of the first conjunct. This is akin to the “Passive Ellipsis Clause Penalty” found in vP-ellipsis (Poppels and Kehler, 2019), where passive ellipsis clauses are degraded regardless of the antecedent clause voice. This observation may have non-trivial implications on the syntax of the DO construction, which are beyond the scope of this study.

## Experiment 2b

Experiment 2b is a replication of Experiment 1b using the dative alternation. The key difference between Experiments 2a and 2b is that Experiment 2a includes the non-elliptical regular coordination sentences as a baseline, to control for any effect general to all coordinate structures.

### Method

#### Participants

In this experiment, participants were 57 self-reported native speakers of English with no vision or hearing disorders. They were recruited through *prolific.co*, an online academic platform. Participants provided informed consent and were compensated at a rate of approximately $10 per hour.

#### Materials

Since Experiment 2a found that gapping is degraded when the second conjunct involves the *DO*-frame, we tested only sentences with *PP*-frame second conjunct argument structure in Experiment 2b. A total of 16 critical items were each instantiated as four conditions in a 2 × 2 factorial design, with *argument structure matching* (match vs. mismatch) and the *presence of gapping* (gapping vs. no gapping) manipulated as factors. A list of sample stimuli is shown in (12).

(12) a. Austin promised a banquet to the team, and Sydney a bonus to the crew.
  [Argument structure match/gapping]
  b. Austin promised a banquet to the team, and Sydney promised a bonus to the crew.
  [Argument structure match/no gapping]
  c. Austin promised the team a banquet, and Sydney a bonus to the crew.
  [Argument structure mismatch/gapping]
  d. Austin promised the team a banquet, and Sydney promised a bonus to the crew.
  [Argument structure mismatch/no gapping]

If dative alternation argument structure mismatch is allowed in both gapping and non-elliptical coordination, no effect of *argument structure matching* and no interaction effect of *argument structure matching* and the *presence of gapping* should be expected.

In addition to the critical items, there were 18 grammatical (mean rating = 4.63, SE = 0.09) and 18 ungrammatical fillers (mean rating = 2.49, SE = 0.11) included.

#### Procedure

The same procedure as those of the previous experiments was employed.

### Results

**FIGURE 4:**
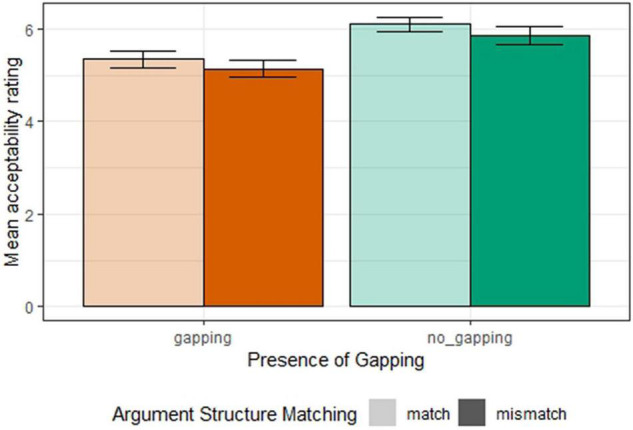
Mean acceptability ratings for all conditions (Experiment 2b).

Figure 4 shows the mean acceptability ratings for all conditions in Experiment 2b. The same ordinal regression model as in Experiment 1b was used to analyze the results. We found a significant effect of *argument structure matching* (β = 0.22, SE = 0.08, *z* = 2.60, *p* < 0.01) and a significant effect of the *presence of gapping* (β = −0.92, SE = 0.17, *z* = −5.49, *p* < 0.001), but no significant interaction between the two factors (β = −0.01, SE = 0.07, *z* = −0.17, *p* = 0.87). This result suggests that the argument structure mismatch penalty does not have a gapping-specific component, contrary to the previous findings in Experiment 1b.

## Discussion

The results of this study present an interesting puzzle. In Experiment 1a, when the two conjuncts of a gapping construction contain the two different argument structures in the locative alternation, the resulting sentence is less acceptable than its counterpart without argument structure mismatch. In Experiment 1b, the argument structure mismatch penalty observed in Experiment 1a is gapping-specific. Although the magnitude of this penalty is small, these observations are still in line with the claim that argument structure mismatch between conjuncts should be ungrammatical in gapping, as proposed by Merchant (2013) among others.

In Experiments 2a and 2b, we adopted the exact same design as those of Experiments 1a and 1b, except that dative alternation was used instead of locative alternation to create argument structure mismatches between conjuncts. In Experiment 2a, there is a marginal argument structure mismatch penalty. Surprisingly, in Experiment 2b, we observed significant main effects of the *presence of gapping* and *argument structure mismatch*, but not their interaction. This suggests that the argument structure mismatch penalty observed in Experiment 2a is general to coordinate structures, elliptical or not. These observations are not expected if argument structure mismatches are ungrammatical due to the identity requirement of gapping.

Although we do not have a definitive answer as to why gapping-specific argument structure mismatch penalty arises with locative alternation verbs, but not with dative alternation verbs, below are three possible explanations.

One possibility is that gapping involves the ellipsis of a category smaller than vP. In this way, argument structure mismatch is not expected to be ungrammatical in gapping: the identity requirement of ellipsis does not affect the little v head in the two conjuncts. But under this approach, not only little v mismatch, but also voice mismatch should be expected to be possible in gapping. This is clearly not the case, given the uncontroversial contrast shown in (2). One potential fix to this problem is to appeal to the parallelism hypothesis of gapping. Previous studies have shown that, during the processing of the gapping construction, the parser, by default, assumes the most parallel analysis of the conjoined structure (Frazier and Clifton, 2001; Carlson, 2002; Kim et al., 2020). Adopting this hypothesis, the contrast in (2) and the gapping-specific argument structure mismatch penalty in Experiments 1a and 1b can be attributed to a gapping-specific processing difficulty. However, it is still unclear why dative alternation argument structure mismatch does not give rise to the same processing difficulty.

Another possible explanation, inspired by the recycling hypothesis (Arregui et al., 2006), is that the DO/PP-frames in the dative alternation are more prone to be misremembered than the *in*-/*with*-frame in the locative alternation. Gapping with DO/PP-frames mismatches is thus ameliorated by a grammaticality illusion. However, this account is unlikely because even in non-elliptical coordination, people find DO/PP-frames mismatches degraded (Experiment 2b), suggesting that they are sensitive to the argument structures of earlier conjuncts rather than misremembering them. Refer to Poppels and Kehler (2019) for more evidence against the recycling hypothesis in vP-ellipsis. A third possibility is that the dative alternation does not involve voice or little v mismatch, whereas the locative alternation and the active-passive contrast do. For example, an analysis can be adopted for dative alternation where the two argument structures are not derivationally related (Pinker, 1989, *inter alia*; Goldberg, 1992, 1995; Krifka, 1999, 2004), while maintaining a derivational analysis for locative alternation and the active–passive contrast. In this way, we can preserve Merchant’s (2013) analysis for gapping. The contrast in (2) and the results from Experiments 1a and 1b can then be attributed to the ungrammatical voice or little v mismatch, and the results from Experiments 2a and 2b would also be expected since dative alternation does not involve little v mismatch. The most obvious drawback of this approach is the inelegance of having a derivational analysis for only locative but not dative alternations, which goes against the traditional Larsonian account where the two are analyzed similarly (Larson, 1988, 2017).

Finally, a reviewer pointed out that the magnitudes of the argument structure mismatch penalties in all of the experiments are small despite statistical significance. It is likely that such small effects may not reflect any grammaticality difference, which is a further challenge to Merchant’s (2008, Merchant, 2013) claims. However, where to draw the line between ungrammatical sentences and degraded yet grammatical sentences is a controversial issue, which we leave for future studies to tackle.

## Conclusion

In sum, this study presents experimental evidence that the locative alternation in gapping gives rise to gapping-specific argument structure mismatch penalty, yet dative alternation does not. This observation challenges analyses of gapping that involve the ellipsis of a category larger than VoiceP, which predicts that gapping should disallow argument structure mismatches in general (Merchant, 2013).

## Data Availability Statement

The raw data supporting the conclusions of this article will be made available by the authors, without undue reservation.

## Ethics Statement

The studies involving human participants were reviewed and approved by the Sungkyunkwan University. The patients/participants provided their written informed consent to participate in this study.

## Author Contributions

NK and JL ran the experiment, conducted the statistical analyses of the data from the study, and supervised the creation of stimuli. Both authors contributed equally to the planning of the research, participated in writing the manuscript and approved the submitted version.

## Conflict of Interest

The authors declare that the research was conducted in the absence of any commercial or financial relationships that could be construed as a potential conflict of interest.

## Publisher’s Note

All claims expressed in this article are solely those of the authors and do not necessarily represent those of their affiliated organizations, or those of the publisher, the editors and the reviewers. Any product that may be evaluated in this article, or claim that may be made by its manufacturer, is not guaranteed or endorsed by the publisher.
